# Annual Meetings of Hospitals

**Published:** 1921-04-02

**Authors:** 


### Annual Meetings of Hospitals.

### The London Homœpathic Hospital.

Major-General Lord Cheylesmore announced at the seventy-first annual general meeting of Governors that the Duke of York had consented to become a patron of the hospital. The Earl of Donoughmore, in moving the adoption of the report, said they had been compelled to institute a system of payment by patients. That innovation had been criticised on many grounds, but he did not regard a request for £1 a week as a departure from the voluntary system. The appeal for £7,690 for long deferred repairs and alterations had met with a most generous response. Lord Dysart had promised the last £500, provided the balance was subscribed by July 31. He deplored the fact that the ordinary expenditure for the past year was £8,885 in excess of the income, which, together with the accumulated deficits since 1916, had brought the total deficit to £17,702. He urged the importance of increasing the number of annual subscribers. One of the lessons of the war, he considered, was the importance of small subscriptions.

### Brompton Hospital for Consumption.

Major-General Lord Cheylesmore presided at the annual meeting of Brompton Hospital for Consumption on March 17, when the Committee of Management reported the continued progress of the work of the hospital, the number of patients admitted last year exceeding 2,000. Of that number a very large proportion were ex-Service men, the actual number to date being 3,405. A grant of £3,000 to meet immediate liabilities was received from the King's Fund, and a promise of £7,000 towards the proposal to provide 100 additional beds for ex-Service men. A tentative offer of £180 per bed was also received from the Ministry of Health, but as these two sums together only amounted to half the sum required the Committee fears it will be necessary to postpone the project. Various works and improvements postponed during the war have been completed. The Sanatorium has been entirely redecorated and painted, and a permanent staff engaged in keeping the works and buildings in good condition throughout the year. The cost of providing new electric lifts in place of the hydraulic ones, which have been in constant use for over forty years, was £3,000, and the Committee have sanctioned the expenditure of £1,000 on the purchase of the latest and most modern apparatus for the x-ray department. Improvements in the Nurses' Home would cost about £2,000. The system of requiring contributions towards the cost of treatment of the patients has proved entirely successful. The practice of holding interim courts has been abandoned, and in future meetings will take place annually. By arrangement with the L.C.C., in connection with the scheme for dealing with tuberculosis in London, the hospital will form one of the principle centres for diagnosis where expert opinion is desirable.

### The Hospital for Women, Soho Square.

A letter from the Earl of Shaftesbury, intimating his resignation of the position of President, and nominating Princess Christian as his successor, was read by Earl Winterton, M.P., at the seventy-eighth annual meeting of the Hospital for Women, Soho, which took place recently. The Princess expressed her willingness to become their President. The report presented at the meeting states that the number of in-patients admitted, 1,138, constitutes a record, the previous highest being 1,083 in the year 1914. On several occasions it was necessary to set up emergency beds. The Samaritan Fund was instrumental in sending fifty-five patients to convalescent homes, and spent £176 on surgical instrument and appliances for patients. The Committee of Management expressed their warmest thanks to the Ladies' Association for their generous assistance during the year.

### Poplar Hospital for Accidents.

The sixty-fifth annual report of the Poplar Hospital for Accidents records the gratifying fact that so far the institution has been able to carry on without having to break with its tradition of never being in debt, and expresses the grateful thanks of the Committee to the King's Fund and the Hospital Saturday and Sunday Funds for the increases in the sums allocated to the hospital. The Committee feel justified, with a sum of £29,000 already placed to a suspense account, for the carrying out of the rebuilding scheme, in deciding to commence one section of the scheme early in the current year. The total cost of the scheme is estimated at £54,000, and £42,000 is allotted or promised. The King's Fund has promised £6,000 for the Building Fund from the surplus Red Cross Funds, and £1,000 from their own fund. The sum of £15,000 is still required to complete the scheme. Since the middle of October adult in-patients have been asked to pay 2s. 6d. per day towards the cost of their maintenance. The charge fixed for children between seven and fourteen years of age is 1s. 3d. per day, children under seven free. Total or partial exemption from payment is allowed where necessary.

### Great Northern Central Hospital.

In the unavoidable absence of the Marquis of Northampton, Mr. C. P. Merriam, J.P., presided over the annual Council. He said that last year the institution treated 2,861 in-patients and 179,399 out-patients, as compared with 2,503 and 173,637 respectively in 1919. A number of ex-Service men, for whom the allowance of the Ministry of Health was hardly sufficient, have been cared for. The charges for in-patients were as low as possible, but absolutely necessary. He hoped no one would suppose that they covered the cost to the hospital. They merely assisted its funds. The adjunct of the hospital—its home of recovery—had, during the past year, been moved from "Earlsmead," Hornsey Lane (lent by Mr. E. G. Harrop) to "Summerlee," East Finchley (put at the disposal of the hospital temporarily by Mr. Kennedy Jones, M.P.). "Summerlee," however, was closed in December. "Grovelands," Southgate, had, he was glad to say, been passed on to the hospital as a home of recovery by the Southgate Voluntary Aid Organisation. The total ordinary expenditure during 1920 amounted to £62,000, as compared with £51,856 in the previous year. Possibly an income of from £18,000 to £24,000 would result if insurance patients could be received by the hospital. Mr. Panter, the Secretary, said that the legal business of the amalgamation with the Royal Chest Hospital was proceeding, and would soon be complete. By means of contributors' certificates, now under consideration, contributors to the hospital would, on request, be given certificates to the value of their contributions. Those certificates would be valid for ten years, and the holders thereof, being eligible for and requiring hospital treatment, would receive such treatment up to the value of the certificates held.

### National Hospital for the Paralysed and Epileptic.

The annual meeting was held on March 22. Sir Frederick Macmillan, Chairman of the Board, presided, and stated that the financial position of the hospital has very considerably improved since the last annual meeting. The report recalls the fact that at the last annual meeting it was announced that the main part of the hospital would have to be closed unless a sum of at least £10,000 was raised immediately. Donations received in response to a special appeal amounted to only £1,600, and in June it was decided forthwith to close the in-patients' department. An appeal was made to those who had benefited through the hospital, and a substantial sum was raised. The King's Fund made an emergency grant of £8,000, and the position had so much improved by July that it was decided to keep open all the wards except three. In October the closed wards were reopened, and it was decided to augment the income of the hospital by inviting patients to contribute towards the cost of their maintenance. The total sum received from that source was £16,196. The income for the year amounted to £48,722, and exceeded the expenditure by £8,668. In 1919 the total income was £291,766. The management of the hospital at Bray Court, Maidenhead, which, at the request of the Ministry of Pensions, was established in 1918, has been discontinued by the National Hospital. Over 100 ex-Service men have been able to resume their former occupations or take up self-supporting work, and the hospital has been warmly thanked by the Government for its services. By arrangement with the Ministry of Pensions a separate clinic has been established for ex-Service out-patients. The Home for paralysed ex-Service men at Lonsdale House, Clapham Park, has continued its work throughout the year. The daily average bed occupation was thirty, as against twenty-eight in the previous year. On the closing of St. Marylebone Home for Incurable Women, Weymouth Street, an arrangement was made whereby the Convalescent Home of the hospital at East Finchley received seven remaining patients from the St. Marylebone Home, and took over the balance of the funds of that institution, amounting to between £2,000 and £2,500.

